# Pathology of natural *Francisella tularensis* subsp. *holarctica* infection in two yellow-necked mice (*Apodemus flavicollis*)

**DOI:** 10.1186/s13028-018-0381-9

**Published:** 2018-05-02

**Authors:** Gete Hestvik, Henrik Uhlhorn, Roland Mattsson, Eva Westergren, Fredrik Södersten, Sara Åkerström, Dolores Gavier-Widén

**Affiliations:** 1 Department of Biomedical Sciences and Veterinary Public Health, Faculty of Veterinary Medicine and Animal Sciences, Swedish University of Agricultural Sciences (SLU), P.O. Box 7028, 75007 Uppsala, Sweden; 2 Department of Pathology and Wildlife Diseases, National Veterinary Institute (SVA), 75189 Uppsala, Sweden; 3 Department of Microbiology, National Veterinary Institute (SVA), 75189 Uppsala, Sweden

**Keywords:** *Apodemus flavicollis*, *Francisella*, Immunohistochemistry, Pathology, Tularemia, Small rodent, Yellow-necked mouse

## Abstract

**Background:**

Tularemia is a zoonosis caused by the bacterium *Francisella tularensis*. It has a wide host range, which includes mammals, birds and invertebrates. *F. tularensis* has often been isolated from various species of small rodents, but the pathology in naturally infected wild rodent species has rarely been reported.

**Case presentation:**

Herein, we describe the pathology of tularemia in two naturally infected wild yellow-necked mice (*Apodemus flavicollis*). To visualize *F. tularensis* subsp. *holarctica*, indirect immunofluorescence and immunohistochemistry were applied on tissue sections. Real time polymerase chain reaction detected the bacterium in samples from liver and spleen in both mice. The only finding at necropsy was splenomegaly in one of the mice. Histological examination revealed necrotic foci in the liver associated with mild inflammation in both mice. Immunohistochemistry and indirect immunofluorescence showed bacteria disseminated in many organs, in the cytoplasm of macrophages, and intravascularly.

**Conclusions:**

The two yellow-necked mice died of an acute disease caused by tularemic infection disseminated to many organs. Further investigations of naturally infected small rodents are important to better understand the variability in pathological presentation caused by infection by *F. tularensis* subsp. *holarctica*, as well to elucidate the importance of small rodents as transmitters and/or reservoirs.

## Background

Tularemia is a zoonotic infectious disease caused by the bacterium *Francisella tularensis*. *F. tularensis* has several subspecies, but only *F. tularensis* subsp. *holarctica* on the Northern Hemisphere, and *F. tularensis* subsp. *tularensis*, in North America, are known to cause disease [1]. *F. tularensis* subsp. *tularensis* is more virulent, causing more severe disease compared to subsp. *holarctica*. The bacterium has a wide host range, including mammals, birds and invertebrates [2, 3]. The severity of disease upon infection varies between species. For example, many small rodent species, such as *Apodemus* spp. and mountain hares (*Lepus timidus*) contract an acute, severe disease rapidly leading to death while carnivores are quite resistant and develop no or mild disease [4]. Infection with *F. tularensis* occurs through direct contact with infected tissues and fluids, via mucous membranes, inhalation and ingestion. Indirect infection is common, through bites from arthropod vectors, in particular mosquitoes, biting flies, and ticks. [5]. Tularemia is endemic in parts of Sweden, frequently affecting mountain hares and European brown hares (*Lepus europaeus*) [6]. There are several studies regarding detection of *F. tularensis* in organs and seroprevalence in wild small rodents in the former Soviet Union and Europe [7, 8], but publications of pathological manifestations in wild rodents are scarce. The pathology in small rodents has been investigated in experimental infections with *F. tularensis* subsp. *holarctica* [9–11], but studies of the pathology in naturally infected wild small rodents are few. One study describes the pathology in field voles (*Microtus agrestis*) in Finland, and another the pathology in house mice (*Mus musculus domesticus*) in Switzerland [11, 12]. Here we describe the pathology in two naturally infected yellow-necked mice (*Apodemus flavicollis*).

## Case presentation

Two adult male yellow-necked mice found dead in a courtyard in Södertälje, central-eastern Sweden, in September 2010 were sent to the National Veterinary Institute (SVA) for necropsy.

To detect *F. tularensis* subsp. *holarctica,* quantitative real time polymerase chain reaction (qPCR) was applied on swab samples from liver and spleen pooled for each mouse separately. Tissue samples were swabbed using sterile cotton swabs, a sampling method used for routine diagnostics for many years at our laboratory. The swabs were incubated in 570 μL G2 buffer and 30 μL proteinase K solution (EZ1 Tissue DNA Extraction Kit) (Qiagen, Sollentuna, Sweden) at 56 °C for 15 min under continuous agitation followed by incubation at 95 °C also under continuous agitation for 15 min. An EZ1 Tissue DNA Extraction Kit and the EZ1 Advanced Instrument (with the Bacteria Card) (Qiagen, Sollentuna, Sweden) was used for extracting DNA. For the DNA extraction, 195 µL of the above lysate plus 5 µL of internal positive control (IPC, virions from seal herpes) were used. The extracted DNA was eluted in 50 µL elution buffer, and 2 μL of the 50 μL elution buffer was used as template for the qPCR. The qPCR was based on a specific point mutation, SNP, which are inherited in the strains belonging to *F. tularensis* subsp. *holarctica*. The primers used for target FtB15 were following, FtB15-F: CCATCAGCAGTAGTATAACCACCAA, FtB15-R: TGGCGCAGATATGACTAAAGTC. Probe for FtB15: TATTACTAGGAATGGCGCGC (FAM-MGB). The primers used for the internal positive control (IPC) were following, IPC-F: GGGCGAATCACAGATTGAATC, IPC-R: GCGGTTCCAAACGTACCAA, probe: TTTTTATGTGTCCGCCACCATCTGGATC (Cy5-Taqman probe BHQ-2). As a positive control we used DNA from a *F. tularensis* subsp. *holarctia* type B strain FSC200. A 45 cycle qPCR fast program was used.

At necropsy, tissue samples from liver, spleen, kidney, lung and heart were fixed in 10% neutral buffered formalin and routinely processed for histopathology. Tissue sections were cut at 4 µm and stained with hematoxylin and eosin (HE).

To detect and visualize *F. tularensis* in tissue sections, indirect immunofluorescence (IIF) and immunohistochemistry (IHC) were applied.

IIF, a method previously used in routine diagnostics at SVA, was performed on direct smears of bone marrow, and on unstained formalin fixed tissue sections of liver, spleen, lung and kidney, applying an in-house *F. tularensis*-positive rabbit serum (polyclonal antibodies) (SVA, Uppsala, Sweden) for detection. Sections were incubated in a 37 °C moist-chamber for 30 min with the rabbit primary polyclonal antibody diluted 1:20 in 0.05 M phosphate buffer, pH 7.9 (PBS). After rinsing the slides in PBS, a secondary fluorescein-labeled goat anti-rabbit IgG (H+L) (Vector, Laboratories, Burlingame, CA, USA) diluted 1:20 in PBS was applied. The slides were incubated in a 37 °C moist-chamber for 30 min, rinsed in PBS and mounted with a cover glass and phosphate-buffered glycerol, pH 8.6. To exclude false negative staining, sections of liver and spleen from an experimentally infected laboratory mouse was examined in the same way. To exclude false positive staining, serum from a known tularemia-negative rabbit was used as primary antibody. The slides were examined in a fluorescence microscope, excitation at 490 nm and emission at 530 nm. Bright green fluorescence and a morphology consistent with the bacterium was considered as positive.

IHC was performed on sections from all histopathologically examined organs using a mouse primary monoclonal antibody, FB11 (Meridian Life Science Inc., Nordic Biosite AB, Täby, Sweden) directed against *F. tularensis* lipopolysaccharide antigen. To enable detection of *F. tularensis* in mouse tissue using a mouse primary antibody, Vector^®^M.O.M™ (Vector Laboratories) was also applied to reduce endogenous mouse Ig staining. Sections were incubated in the primary antibody diluted in M.O.M Diluent at 1:3000 for 30 min, followed by incubation in a working solution of M.O.M. biotinylated Anti-Mouse Ig Reagent for 10 min. The detection system used was VECTASTAIN^®^elite ABC standard kit (Vector Laboratories,), together with the peroxidase substrate DAB (Dako, Agilent, Glostrup, Denmark). Counterstaining was performed with Mayer´s hematoxylin. A serial parallel section of each IHC-tested section was incubated with 2% bovine serum albumin (BSA) instead of the primary antibody, which served as a primary antibody-omit negative control. A known tularemia-positive liver sample from a European brown hare, in which *F. tularensis* subsp. *holarctica* previously had been detected by q-PCR, was included as positive control.

**Fig. 1:**
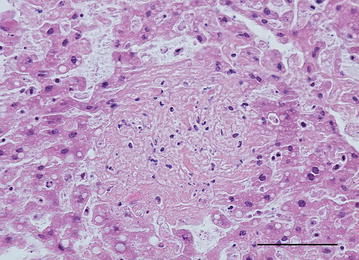
Photomicrograph showing liquefactive necrosis in the liver. Nuclear debris is seen as dark basophilic granules in the lesion. Yellow-necked mouse, HE. Bar = 100 µm

**Fig. 2:**
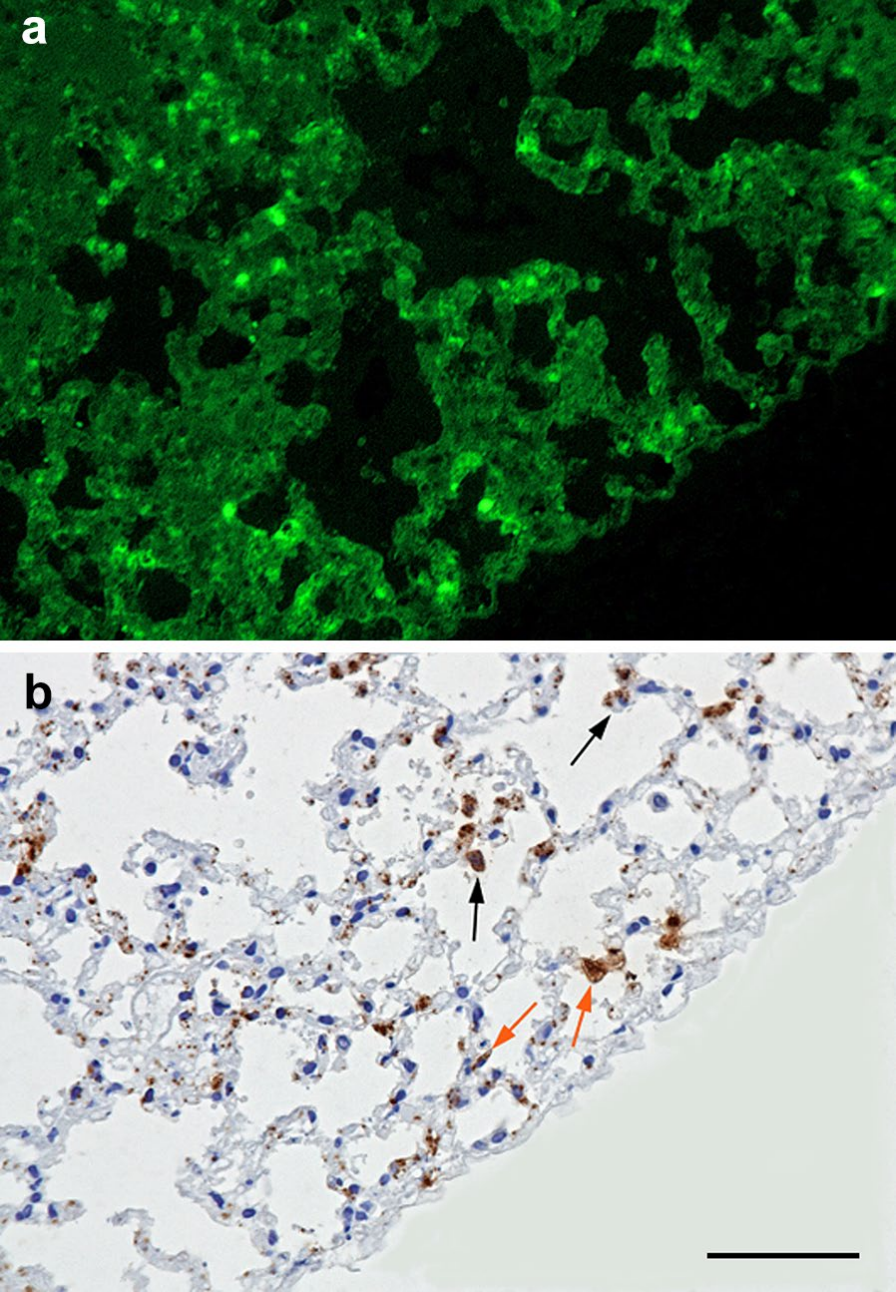
Microcopic visualization of *F. tularensis* in the lung of a yellow-necked mouse. **a** Indirect immunofluorescence photomicrograph of the lung infected by *F. tularensis*. *F. tularensis* bacteria are visualized in bright green fluorescence in the alveolar septa. **b** Immunohistochemistry for *F. tularensis* of the same area shown in **a** reveals their presence in the cytoplasm of pneumocytes (orange arrow) and alveolar macrophages (black arrow). Yellow-necked mouse. Bar = 100 µm

**Fig. 3:**
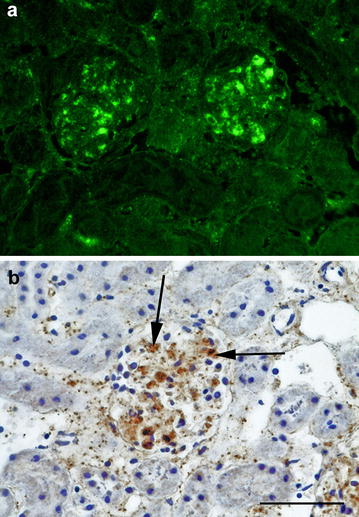
Indirect immunofluorescence photomicrograph of a kidney infected by *F. tularensis*. **a** Indirect immunofluorescence showing *F. tularensis* (bright green fluorescence) in the glomeruli. **b** Immunohistochemistry showing the location of the bacteria in glomerular capillaries (black arrow). Yellow-necked mouse. Bar = 50 µm

The two yellow-necked mice were in poor body condition and had marked postmortem changes. Apart from slight splenomegaly in one mouse, no other gross changes were observed. Histological examination revealed numerous necrotic foci in the liver in one mouse and few in the other (Fig. 1). Some of the necroses were coagulative showing necrotic hepatocytes with preserved architecture in the periphery of lesions, but most were lytic. Mild infiltration of lymphocytes, plasma cells and macrophages was seen in association to the necrotic areas, and lymphocytes could also be seen in the sinusoids. Autolysis was severe in the spleens, but there was a strong suspicion of necrosis. In one mouse, pulmonary alveolar septa were moderately to markedly thickened and multifocally consolidated by lymphocytes, plasma cells, macrophages and a few heterophils. Multifocal necrosis scattered in the inflamed tissue was suspected but was difficult to assess with certainty because of autolysis. No histopathological changes could be seen in the heart or kidney. IIF showed bacteria disseminated in liver, spleen, bone marrow, lung and kidney, in lesions or intravascularly (Figs. 2a, 3a). IHC visualized *F. tularensis* intravascularly in glomerular capillaries and interstitial blood vessels in the kidney, in liver sinusoids, and in larger vessels in the liver and heart. In the lung, bacteria were detected in the cytoplasm of alveolar macrophages as well as in pneumocytes, alveolar septal capillaries and larger blood vessels (Figs. 2b, 3b). qPCR detected *F. tularensis* subsp. *holarctica* in pooled samples from the liver and spleen in both mice. The compiled result show that both mice died of acute disseminated septic tularemia.

## Discussion and conclusions

The pathological findings in the yellow-necked mice were similar to those described in studies of laboratory mice [9], wild common hamsters (*Cricetus cricetus*) [10], and field and bank voles (*Myodes glareolus*) [11], all experimentally infected with *F. tularensis* subsp. *holarctica*.

Gross lesions reported in laboratory mice were enlarged spleens and mottled, pale livers. Microscopically, necrosis was evident in the organs examined; spleen, liver and lung. The only gross lesions in the hamsters were enlarged spleens with grossly visible necrosis. In the voles, no gross changes were found. In hamsters, field and bank voles, histopathology showed necroses in spleen, liver, bone marrow and lymph nodes. The hamsters also had acute nephrosis and interstitial bronchopneumonia. IHC revealed *F. tularensis* intravascularly in several organs of the hamsters and voles.

In a study of naturally infected wild field voles [11], extensive necrosis was evident in the spleen, whereas in the liver only single cell necrosis was seen. Bacteria were found in vessels and capillaries of the lung, liver, spleen and kidney, shown by IHC. In naturally infected free-ranging house mice in Switzerland, necrosis in spleen, liver and lung were found. The lung lesions had variable degree of severity [12].

In the literature, it is frequently discussed whether small rodent species die of acute disease or if they may act as reservoirs or transmitters of the bacteria. The term rodents refer to mammals of the order *Rodentia*, which is the greatest diversified mammalian order, containing 40% of all mammal species inhabiting a broad range of different habitats [13]. There is a high diversity of biological characteristics among the rodent species, and therefore the sensitivity to develop disease might differ between them [14]. The majority of studies performed on wild small rodents have been limited to detection of *F. tularensis* in organs and/or of antibodies but have not included reports of pathology.

The two yellow-necked mice included in our study were in poor body condition, had necroses and mild inflammation in several organs, as well as intravascular bacteria. The mice survived long enough to develop subacute lesions and probably died of sepsis. The very limited existing data on the pathobiology of the infection makes it difficult to assess the epidemiological role of small rodents as, for example, possible natural reservoirs and transmitters of *F. tularensis* subsp. *holarctica*. The knowledge gaps of natural infection in small rodents include the range and variability in clinicopathological presentation and the distribution and abundance of bacteria in organs and excretions to asses shedding patterns. Potentially, chronically infected small rodents could act as spreaders or carriers of infection, but such a chronic form of infection has not yet been reported. Nonetheless, several small rodent species, such as lemmings (*Lemmus* spp.), house mice, yellow-necked mice and black rats (*Rattus rattus*) have been implicated in the epidemiology of tularemia in humans and animals [15, 16]. For example, oropharyngeal tularemia has been attributed to consumption of water from water wells contaminated by tularemic lemmings [16] and pneumonic forms of tularemia to inhalation of hay contaminated by tularemic rodents [17]. More investigations on the pathobiology of natural tularemia infection in rodents is warranted to provide information on organ lesions and shedding patterns, and to investigate the role of wild small rodents as potential transmitters and/or reservoirs.
